# Application of Reductive ^13^C-Methylation of Lysines to Enhance the Sensitivity of Conventional NMR Methods

**DOI:** 10.3390/molecules18067103

**Published:** 2013-06-18

**Authors:** Tanmay S. Chavan, Sherwin Abraham, Vadim Gaponenko

**Affiliations:** 1 Department of Medicinal Chemistry, University of Illinois at Chicago, 900 S Ashland, Chicago, IL 60607, USA; E-Mail: tchava2@uic.edu; 2 Department of Molecular and Cellular Physiology, Stanford University, Stanford, CA 94305, USA; E-Mail: sherwinjcb@gmail.com; 3 Department of Biochemistry and Molecular Genetics, University of Illinois at Chicago, 900 S Ashland, Chicago, IL 60607, USA

**Keywords:** K-Ras, calmodulin, reductive methylation, hypervariable region, NMR

## Abstract

NMR is commonly used to investigate macromolecular interactions. However, sensitivity problems hamper its use for studying such interactions at low physiologically relevant concentrations. At high concentrations, proteins or peptides tend to aggregate. In order to overcome this problem, we make use of reductive ^13^C-methylation to study protein interactions at low micromolar concentrations. Methyl groups in dimethyl lysines are degenerate with one ^13^CH_3_ signal arising from two carbons and six protons, as compared to one carbon and three protons in aliphatic amino acids. The improved sensitivity allows us to study protein-protein or protein-peptide interactions at very low micromolar concentrations. We demonstrate the utility of this method by studying the interaction between the post-translationally lipidated hypervariable region of a human proto-oncogenic GTPase K-Ras and a calcium sensor protein calmodulin. Calmodulin specifically binds K-Ras and modulates its downstream signaling. This binding specificity is attributed to the unique lipidated hypervariable region of K-Ras. At low micromolar concentrations, the post-translationally modified hypervariable region of K-Ras aggregates and binds calmodulin in a non-specific manner, hence conventional NMR techniques cannot be used for studying this interaction, however, upon reductively methylating the lysines of calmodulin, we detected signals of the lipidated hypervariable region of K-Ras at physiologically relevant nanomolar concentrations. Thus, we utilize ^13^C-reductive methylation of lysines to enhance the sensitivity of conventional NMR methods for studying protein interactions at low concentrations.

## 1. Introduction

Ras proteins belong to a large family of GTPases that play an important role in the transmission of extracellular stimuli to intracellular signaling cascades. The most well studied of these are the MAP kinase and Akt/mTOR pathways. Ras proteins cycle between GTP-bound active and GDP-bound inactive states. Ras proteins contain the N-terminal catalytic domain that binds GTP or GDP and the C-terminal hypervariable region (HVR). Effector recruitment occurs in the GTP-bound state, while HVR undergoes a series of post-translational modifications with lipids and a methyl group that influence Ras activity. These modifications are essential for membrane localization and protein-protein interactions. 

The important members of Ras family include H-, N- and K-Ras. Activating mutations in Ras genes are found in nearly 30% of all human cancers [1]. Of these, K-Ras mutations predominate, accounting for more than 85%. Mutations in K-Ras4B, a splice variant of K-Ras, occur in up to 90% of pancreatic cancers [2,3], 57% of colorectal cancers [4] and 50% of lung cancers [1,5,6]. Moreover, autosomal mutations in K-Ras are associated with Noonan syndrome, which is a developmental disorder [7,8]. K-Ras4B is also indispensible for embryonic development [9,10] and deletion of K-Ras in mouse embryos results in lethal cardiac, neurological, hematopoetic and liver defects [11]. 

One such interaction is observed with the calcium-modulated protein calmodulin (CaM). CaM binds K-Ras4B and influences its downstream activity in a calcium dependent manner [12]. We recently identified the HVR as the site responsible for the binding affinity and specificity of CaM to K-Ras4B [13]. The HVR of K-Ras4B is unique among Ras proteins because it contains a polylysine region and a farnesyl group. Other Ras proteins are palmitoylated in addition to undergoing farnesylation. The importance of the polylysine region and farnesyl moiety for K-Ras4B-CaM binding has been suggested by Lopez-Alcala *et al*. [14]. The structure of CaM contains two lobes comprising the N and C terminal domains, which are connected by a linker region. Binding of CaM to other proteins induces a conformational change in CaM. Many structural studies of CaM binding to its target enzymes use only CaM binding domains (CBDs) [15,16,17]. CBDs serve as an excellent model for interactions of fully intact enzymes with CaM. Calcium loaded CaM binds and dissociates K-Ras4B from the membrane in breast cancer cell lines (MCF-7). Importantly, CaM has recently emerged as a key regulator of K-Ras4B in response to platelet derived growth factor (PDGF) signaling [18]. K-Ras4B-CaM interactions also regulate cell cycle progression through the ERK2 pathway [19]. Furthermore, CaM can prevent Ras activation by PKC in fibroblasts [20]. In response to glutamate, CaM causes reversible intracellular translocation of K-Ras4B in hippocampal neurons [21]. 

In this report we investigate binding between the post-translationally lipidated hypervariable region (HVR) of a human proto-oncogenic GTPase, K-Ras, and CaM. We use a combination of biochemical methods including isothermal titration calorimetry (ITC), dynamic light scattering (DLS), fluorescence, and nuclear magnetic resonance (NMR) to examine this interaction in detail. In order to study a more relevant binding we introduced post-translational modifications in the HVR domain. We discovered that fully post-translationally modified HVR (FM-HVR) aggregates at concentrations higher than 10 μM. In order to study its binding to CaM, protein concentrations lower than 10 μM are desirable. Conventional HSQC NMR is not sufficient to study proteins at such low concentrations. To study binding at a concentration where FM-HVR does not aggregate, the reductive methylation technique was applied to CaM to identify the domains responsible for binding to FM-HVR. Reductive methylation introduces two ^13^C methyl groups on lysine residues of the protein. The methyl groups in dimethyl lysines are degenerate and result in one ^13^CH_3_ signal arising from two carbons and six protons, as compared to one carbon and three protons in aliphatic amino acids. This leads to an intense signal, which can be detected even at low protein concentrations. In addition, increased sensitivity may be related to superior relaxation properties of methyl groups on lysines that result from their commonly low order parameters [22]. This is not possible using conventional ^15^N and ^13^C labeling. We detected NMR signals at concentrations as low as 650 nM. When compared to the chemical shift perturbations obtained by carrying out the same experiment at higher concentrations, we observed that the non-specific binding produced a different spectrum. Thus, we have shown that specific binding of FM-HVR is different from the non-specific aggregate binding to CaM and have provided evidence for the use of reductive methylation as a tool to study protein-protein interactions at low micromolar to nanomolar concentrations.

## 2. Results and Discussion

### 2.1. ^15^N-HSQC Titration of ^15^N-CaM with FM-HVR does not Reveal a Continuous Binding Interface

**Figure 1:**
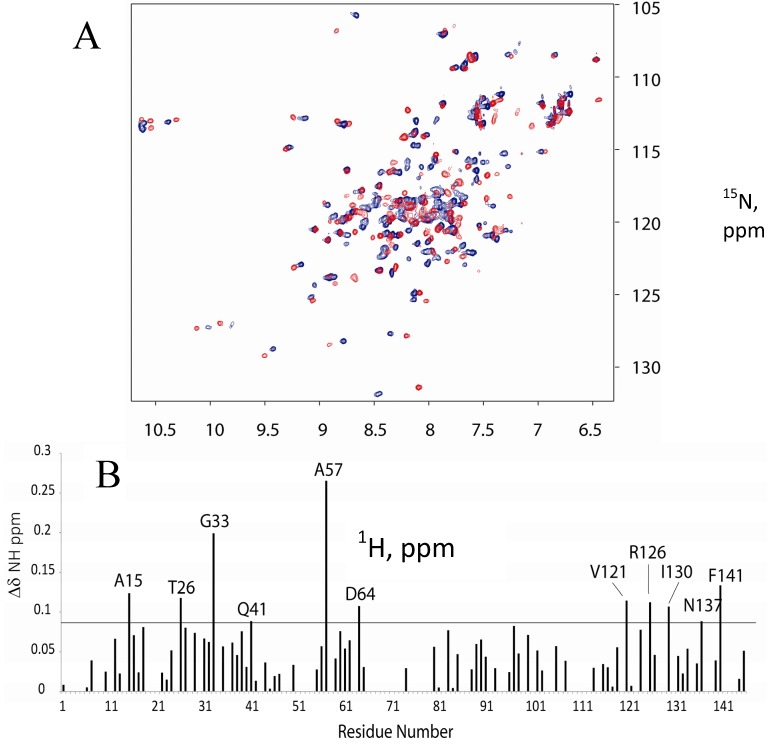

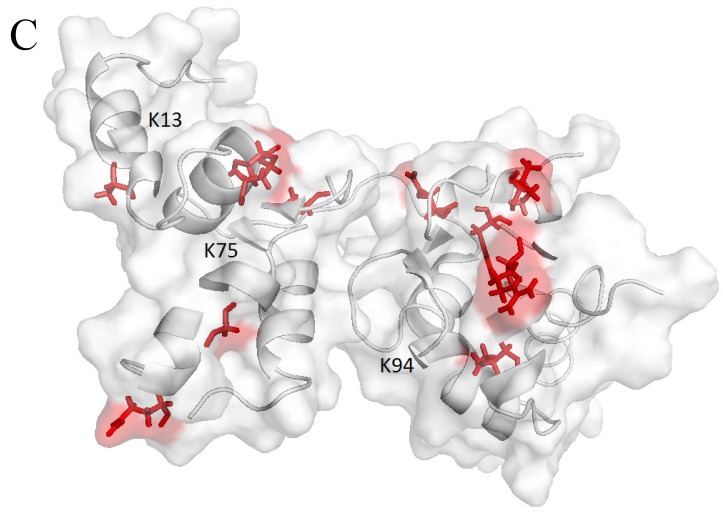
(**A**) Overlay of CaM spectrum without (red) and with (blue) the FM-HVR peptide. The final concentrations of CaM and FM-HVR were 25 μM and 50 μM, respectively. (**B**) Residual chemical shifts obtained after the NMR titration of CaM with FM-HVR peptide. The horizontal line in the graph shows the sum of average chemical shift perturbation and standard deviation. Residues that show zero chemical shift difference on the graph were the ones that were not assigned due to resonance overlap. (**C**) Statistically significant chemical shift differences were mapped on the structure of CaM (PDB: 1CFF). K13, K75 and K94 have been labeled. We make the use of these residues in reductive methylation to investigate CaM FM-HVR interactions.

The conventional method for determination of protein binding interfaces relies on recording ^15^N- HSQC spectra of ligand-bound proteins at different ligand concentrations. To investigate the binding interface between the FM-HVR peptide and CaM, ^15^N-, ^1^H-NMR spectra of the CaM titrations with the peptide were recorded. A solution of CaM was titrated in FM-HVR such that their final concentrations were 25 μM and 50 μM, respectively. Figure 1A,B show an overlay of the titration spectra and a graph of the chemical shift perturbations induced by peptide binding, respectively. Residues (A15, T26, G33, Q41, A57, D64, V121, R126, I130, N137, and F141) in all the three domains show statistically significant chemical shift perturbations. Figure 1C shows the results of chemical shift perturbations mapped on the three-dimensional structure of CaM and highlights the discontinuous binding interface formed by FM-HVR. The fact that several binding sites are observed suggests the presence of non-specific interactions. Moreover, since the concentration of FM-HVR was high, it is possible that the observed binding events are caused by non-specific interactions of the aggregated peptide with CaM. 

### 2.2. Farnesylation and Methylation of HVR Cause Aggregation

**Figure 2:**
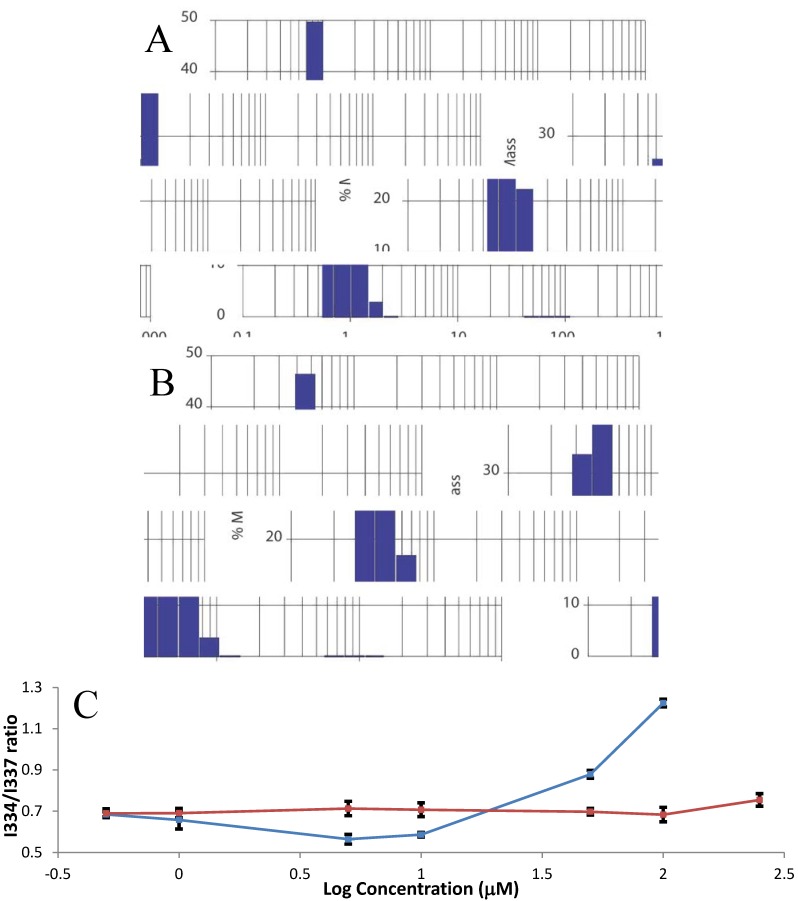
(**A**) Dynamic light scattering of 1mM HVR peptide in PBS at room temperature. (**B**) Dynamic light scattering of 250 μM FM-HVR peptide in PBS at room temperature. (**C**) The plot of fluorescence intensity ratio (I_334_/I_337_) from pyrene excitation spectrum as a function of concentration of modified and non-modified HVR peptides. The FM-HVR peptide, shown in blue, aggregates beyond 10 μM. The HVR peptide, shown in red, does not aggregate.

We then tested the possibility that introduction of hydrophobic farnesyl and methyl groups into HVR induces peptide aggregation. We performed dynamic light scattering experiments on 1 mM HVR and 0.25 mM FM-HVR in PBS at room temperature. The measured hydrodynamic radius for HVR was 1.23 ± 0.05 nm, which corresponded to 3.7 ± 0.46 kDa, shown in Figure 2A. This roughly corresponds to the monomeric form of peptide. However, upon introduction of farnesylation and methylation the hydrodynamic radius of the peptide increased to 8.15 ± 0.46 nm at a four times lower concentration as shown in Figure 2B. This hydrodynamic radius corresponded to a particle of 471.92 ± 63.75 kDa in size. Observation of higher order assemblies of FM-HVR allowed us to conclude that post-translational modifications in K-Ras can lead to aggregation through hydrophobic interactions.

To study physiologically relevant binding of the peptide to CaM we need to know the concentration at which HVR aggregates. To accomplish this, we used a small fluorescent molecule, pyrene, which changes its fluorescent properties upon transitioning from the hydrophilic to the hydrophobic environment. We conducted fluorescence experiments with different concentrations of FM-HVR and HVR. All samples contained 2 μM pyrene. Excitation scans were recorded between 315 to 360 nm with the emission wavelength set at 393 nm. There was a significant 3 nm red shift in the excitation peak of pyrene in the presence of increasing concentrations of FM-HVR. This suggested that pyrene transitioned from the hydrophilic to the hydrophobic environment upon addition of FM-HVR. Conversely, the increasing concentrations of unmodified HVR did not induce similar changes in pyrene fluorescence. Example fluorescence spectra obtained from both the peptides are shown in Figure S7. Determination of the critical aggregation concentration was performed by plotting the intensity ratio I_334_/I_337_ against the logarithm of peptide concentration. The results from our analysis are shown in Figure 2C. The increase in the I_334_/I_337_ ratio of pyrene fluorescence indicates occurrence of FM-HVR aggregation. The graph suggests that aggregation starts in the FM-HVR only after the concentration of 10 μM whereas in HVR samples no aggregation is observed even at 250 μM. Thus, we conclude that the critical aggregation concentration of FM-HVR is 10 μM or greater. We must also acknowledge that there is a slight decrease in the I_334_/I_337_ ratio of the FM-HVR peptide between the concentrations of 1 to 10 μM, however we do not know the exact cause for it. FM-HVR exists in the monomeric and multimeric states. In addition, it is possible that there is a transition intermediate between the monomeric and multimeric states of the peptide. This notion is supported by detection of the peptide species presenting a highly polar environment in the FM-HVR concentration range of 5 to 10 μM. However, at higher peptide concentrations, the hydrophobic forces leading to peptide aggregation dominate and cause an increase in the I_334_/I_337_ ratio of pyrene fluorescence.

### 2.3. Farnesylation Increases Affinity of the Hypervariable Region for CaM

**Figure 3:**
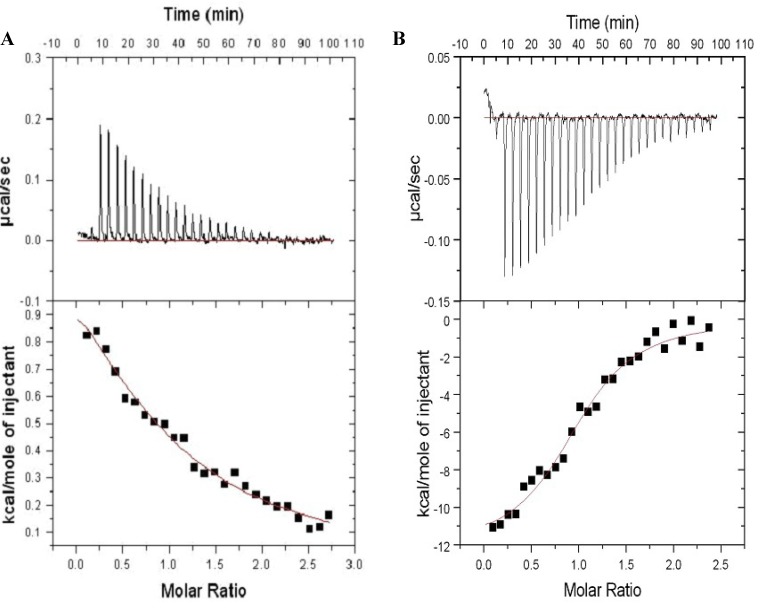
Isothermal Titration Calorimetry of (**A**) 70 μM HVR peptide with 1 mM CaM, and (**B**) 2.9 μM FM-HVR peptide with 33 μM CaM. The upper panels in (**A**) and (**B**) show titration isotherms. The lower panels in (**A**) and (**B**) show the fit of the reference-subtracted titration. The “one set of binding sites” model were used in (**A**) and (**B**).

To characterize the interaction of either FM-HVR or HVR with CaM, ITC experiments were performed wherein CaM was titrated into either FM-HVR or HVR solutions. The farnesylated peptide was at a concentration which does not promote aggregation and non-specific binding. Titration of 1 mM CaM into 70 µM non-farnesylated HVR, as in Figure 3A, yielded a dissociation constant K_d_ = 11.2 ± 1.6 µM, ΔH = 0.65 ± 0.1 kcal/mole, and ΔS = 25.1 cal/deg/mole. The data was fitted with the ‘one set of binding sites’ model. A similar experiment was performed where 33 μM CaM was titrated into 2.9 μM FM-HVR. The data was analyzed using the ‘one set of binding sites’ model. The curve in Figure 3B shows that FM-HVR binds to CaM with an affinity of 0.35 ± 0.06 μM for CaM. The other fitted parameters for this site are ΔH = −14.88 ± 0.99 kcal/mole and ΔS = −19.81 cal/deg/mole. In short, there is almost a 40-fold increase in affinity for CaM when the HVR peptide is farnesylated and methylated, as compared to the non-modified peptide. This suggests that the post-translational modification of HVR is responsible for the increase in affinity to CaM.

### 2.4. ^13^C-HSQC of Reductively Methylated CaM can be Performed at Nanomolar Protein Concentration

To address the problem with non-specific peptide binding to CaM we needed to perform NMR experiments at low protein concentrations. CaM was reductively methylated to increase the sensitivity of NMR signal detection. In order to show that reductively methylated CaM had similar properties to wild-type CaM, we have previously shown that reductive methylation does not affect binding properties of CaM [23]. We also carried out ITC titrations to determine the dissociation constants of the two proteins and they were equal (Figures S4 and S5). All lysines present in CaM are shown in Figure S6.

**Figure 4:**
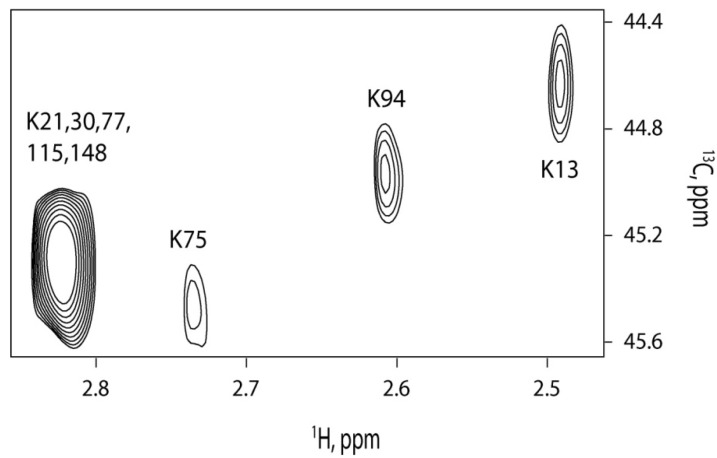
NMR spectrum of 650 nM reductively methylated CaM.

We tested the lowest concentration, at which we could achieve reliable signal detection with reductively methylated CaM. We could observe signals with 650 nM reductively methylated CaM at a signal to noise ratio greater than 10 (Figure 4). The total time for this experiment was 11 hours. The improved sensitivity allows studying protein-protein or protein-peptide interactions at nanomolar concentrations. Signal assignments for reductively methylated CaM were obtained by site directed mutagenesis [23].

**Figure 5:**
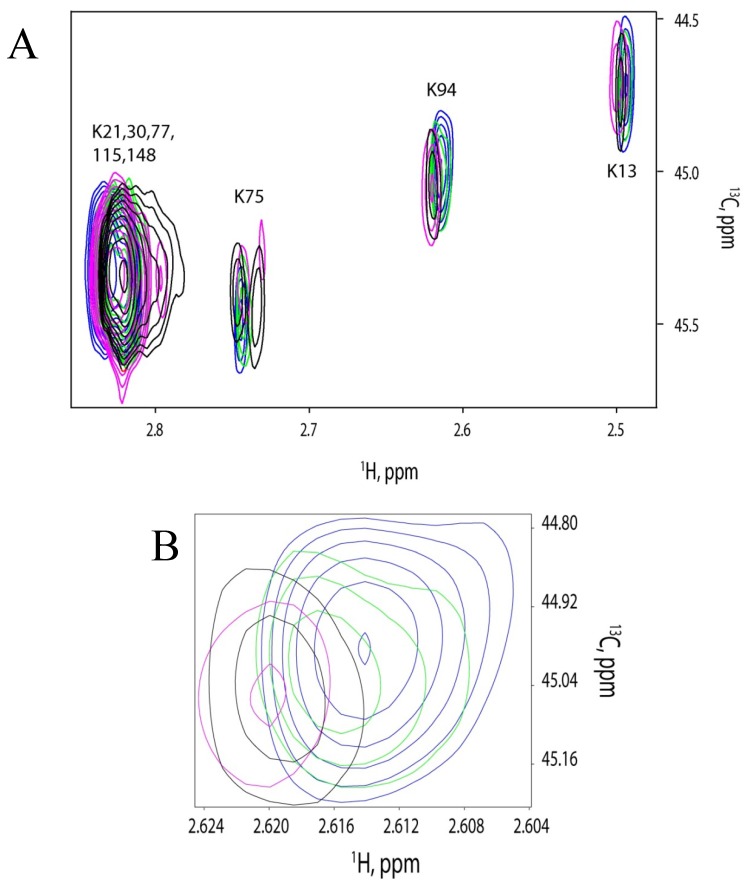

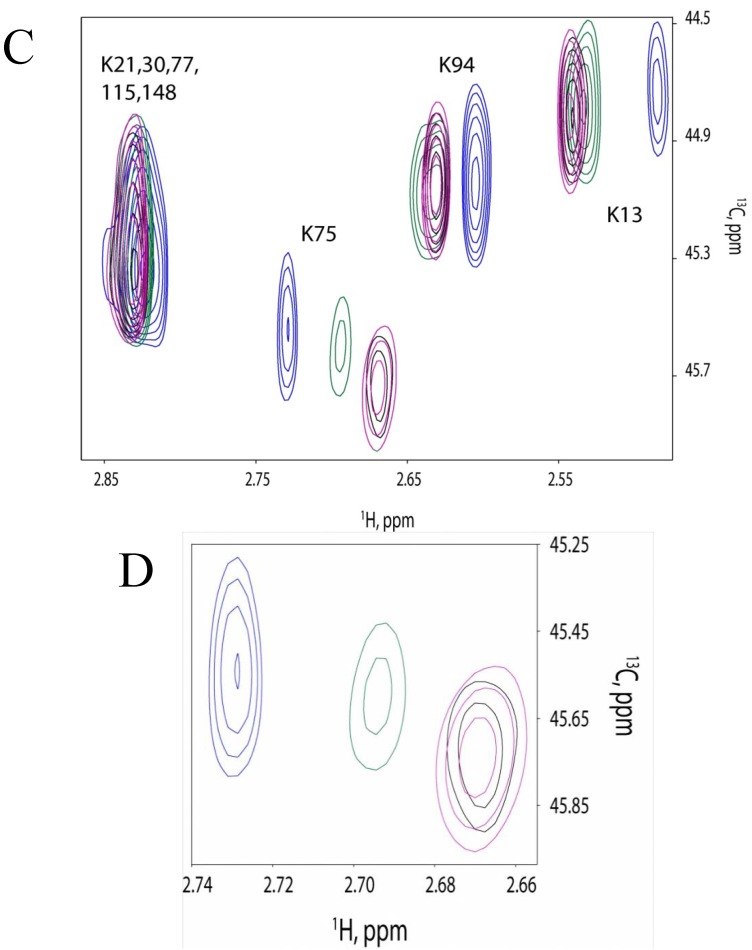
(**A**) NMR titration of different concentrations of FM-HVR into 1uM CaM. Titration ratios were 0:1 (blue), 0.5:1 (green), 1:1 (magenta) and 1.5:1 (black) of FM-HVR and CaM. (**B**) Magnified view of K94 residue showing saturation at 1:1 FM-HVR to CaM ratio. (**C**) NMR titration of different concentrations of FM-HVR into 70uM CaM. FM-HVR concentrations were varied to achieve 0:1 (blue), 1:1 (green), 2:1 (magenta) and 3:1 (black) molar ratios to CaM. (**D**) Magnified view of K75 residue showing saturation at 2:1 FM-HVR to CaM ratio.

For our experiments, we titrated different concentrations (0 μM, 0.5 μM, 1 μM and 1.5 μM) of FM-HVR into 1 μM CaM. This concentration was chosen because of the NMR time-constraints and the absence of peptide aggregation in the low micromolar range. Titration ratios were 0:1, 0.5:1, 1:1 and 1.5:1 of FM-HVR to CaM. It was found that CaM saturated at 1:1 titration ratio as shown in Figure 5A,B. 

The same experiment was carried out using a higher concentration of FM-HVR. CaM concentration in this experiment was 70 µM. FM-HVR concentrations were varied to achieve 0:1, 1:1, 2:1 and 3:1 molar ratios to CaM. This was done to study binding of aggregated FM-HVR to CaM. Figure 5C shows the respective superimposed spectra obtained before and after titrating the various amounts of FM-HVR. We found that FM-HVR saturated CaM at 2:1 titration ratio. This was significantly different from the low concentration saturation event. Comparison of the chemical shifts in Figure 5A,C reveals that the conformation of bound CaM is different for different concentrations of the peptide. These experiments suggest that all the three domains of CaM interact with the modified HVR peptide at high and low concentrations. However, the way CaM binds to aggregates of FM-HVR is significantly different from the way it binds to the monomeric peptide. For example, K75 shows a significant upfield shift in the proton dimension in the spectrum of 70 μM CaM titration, while K75 shows a very minor change in the spectrum of 1 μM CaM titration.

### 2.5. Discussion

The role of farnesyl modification in lipid binding has received significant attention. Recent progress solving the crystal structure of farnesylated GTP-bound protein Rheb in complex with phosphodiesterase suggests that the farnesyl moiety participates in low specificity protein-protein interactions [24]. However, more studies on other proteins are needed to demonstrate the importance of farnesylation in protein-protein interactions.

Generally, protein lipidation is associated with increased binding affinity. There are many literature reports supporting the idea that post-translational lipidation of proteins causes their intracellular trafficking and binding effector molecules [25,26,27]. In certain cases, stability of these proteins is attributed to their post-translational modifications [28].

A majority of small G-proteins undergo requisite post-translational farnesylation at their COOH-terminal cysteine residues. This modification controls subcellular localization and facilitates interactions with effector proteins. One of the prominent targets of farnesylation in humans is a p21 GTPase, K-Ras4B. Our research demonstrates that K-Ras4B farnesylation increases the binding affinity for CaM. This is consistent with previously reported observations. For instance, Lopez-Alcala *et al*. showed that a non-farnesylatable mutant of Ras did not bind CaM [14]. In addition to increasing protein binding affinity, we also observe that farnesylation of K-Ras4B alters the mode of protein-protein interactions. We previously reported that the HVR of K-Ras4B constitutes the major binding site for CaM. Unmodified HVR predominantly binds to the C-terminal domain and the linker region of CaM. Surprisingly, post-translational modifications in HVR engage the N-terminal domain of CaM. Thus, when the HVR is modified, all three domains of CaM participate in K-Ras4B binding. This is shown by recording the NMR spectra of titrations of FM-HVR and CaM. During our experiments with FM-HVR we discovered that the data interpretation is complicated by the fact that FM-HVR aggregates. Nonspecific peptide-protein interactions were observed at peptide concentrations greater than 10 μM.

Protein aggregation often limits the use of NMR for analysis of ligand binding to proteins. Sensitivity issues prevent the use of NMR spectroscopy at physiologically relevant protein concentrations that are often in the nanomolar range. To address this limitation we used reductive methylation of lysine residues in the protein of interest. Given the fact that protein aggregation is a common phenomenon promoted by millimolar protein concentrations required for NMR, reductive methylation provides a viable method to study binding events at physiologically relevant concentrations. 

Using reductively methylated CaM we were able to acquire a ^13^C-HSQC spectrum at a concentration as low as 650 nM. Subsequent NMR titration experiments at 1 and 70 μM CaM with FM-HVR produced different binding interfaces and different binding affinities. At low CaM concentration we observed appearance of new signals in the N- and C-terminal domains as well as the linker region upon addition of FM-HVR. The saturation of the FM-HVR binding site in CaM occurred at 1:1 CaM to peptide molar ratio. The dissociation constant determined by ITC is 350 nM. This value is in similar to the previously published dissociation constant for binding of farnesylated K-Ras to CaM [29]. Conversely, when we titrated 70 μM CaM with FM-HVR we observed chemical shift changes in ^13^CH_3_ (13, 94, 75, 21, 30, 77, 115,148) groups of lysines in CaM. The most dramatic chemical shift perturbations occurred in the linker region of CaM. Although the N- and C-terminal domains and the linker region of CaM were involved in FM-HVR binding, the binding interface was clearly different from that observed at 1 μM CaM. The saturation of CaM with FM-HVR occurred at 1:2 molar ratio of CaM to peptide. This is indicative of significantly lower binding affinity of CaM to aggregated FM-HVR as compared to the affinity between CaM and FM-HVR monomers. These observations allowed us to conclude that physiologically relevant FM-HVR binding to CaM occurs in the nanomolar range.

The technique of reductive methylation is widely used in x-ray crystallography and is found not to affect protein structure or to introduce spurious protein-protein interactions [30]. Reductive methylation does not completely remove the positive charge in lysine epsilon amino groups and, therefore does not induce hydrophobic surfaces in proteins. Enhanced sensitivity is very beneficial for NMR experiments at low protein concentrations. We have used HSQC experiments in our studies. However, the sensitivity can be further improved by the use of experimental schemes like SO-FAST HMQC that relies on standard data sampling in the indirect dimension and allows fast and sensitive NMR data acquisition [31].

Although we believe that reductive methylation has great potential for NMR investigation of protein-protein interactions at physiologically relevant concentrations, we caution that reductive methylation may not be universally applicable to all proteins. Some proteins can be inactivated by reductive methylation. For example, myo-inositol monophospatase has been found to lose its catalytic activity after reductive methylation [32]. In our case, reductive methylation of CaM does not affect calcium loading or antagonist binding properties [23]. Moreover, we also show that the dissociation constants of the interaction between reductively methylated CaM or unmodified CaM with FM-HVR are similar.

One other possible issue with the use of reductive methylation in NMR is related to the limited chemical shift dispersion for methyl groups [33,34]. This is due to the fact that lysine side chains tend to be solvent exposed. In this state, the methyl groups do not experience significant variation in their electromagnetic environment that is needed for chemical shift dispersion. We previously found that the uniform influence of the solvent on chemical shifts can be overcome by adjusting the pH closer to the pKa of lysine side chains. Under these conditions, chemical shifts reflect variability in lysine pKa values [23]. At the same time, dimethyl lysines may exhibit lower spectral dispersion as compared to monomethyl lysines [35]. Although monomethylated lysines have previously been found to provide improved resolution in comparison with the dimethylated variant, we found signals for dimethylated lysines in CaM sufficiently resolved. Moreover, as we show that the dimethyl lysine-modified CaM interacts with FM-HVR in similar manner to non-methylated CaM. Finally, post-translationally introduced ^13^C methyl groups represent very small spin systems. This property prevents the use of sequential assignment techniques. To address this issue, using CaM we showed that signal assignments in reductively methylated NMR samples can be obtained using site-directed mutagenesis [23]. In conclusion, we have uncovered complex binding behavior imparted by post-translational modifications on K-Ras4B. According to our model, farnesylation increases the binding affinity of the protein and alters the way other protein molecules interact with K-Ras4B. 

## 3. Experimental

### 3.1. Isothermal Titration Calorimetry (ITC)

Titration isotherms were obtained using VP-ITC microcalorimeter (MicroCal LLC, Northampton, MA, USA) at 25 °C. For the experiments, CaM, FM-HVR and non-modified HVR peptides were dialyzed against 50 mM Tris-citrate pH 6.5, 50 mM NaCl, 5 mM MgCl_2_, 1 mM TCEP and 10 mM CaCl_2_. The peptide (FM-HVR or HVR) solution was always taken in the cell of volume 1.4 mL and CaM was always titrated using a syringe in twenty-eight 10 µL injections preceded by one 2 µL injection to account for diffusion artifacts. The reference data was obtained by titrating CaM into buffer. This reference data was subtracted from the actual peptide titration data. Analysis of the data was done using Origin 7.0 software. Fitting was done using the “one set of sites” model or “two sets of sites” model. The other thermodynamic parameters were also obtained using the fitting procedures.

### 3.2. Particle Size Analysis

For studying aggregation of peptides, dynamic light scattering experiments were performed on a DynaPro-801 molecular sizing instrument (Wyatt Technology Europe, Dernbach, Germany). The peptide samples were dialyzed against PBS. The sample volume of 250 μL was used for the experiments. The FM-HVR and HVR peptide concentrations were 250 μM and 1 mM, respectively. Samples were centrifuged at 12000 rpm for 10 min before experiments. Analysis of the data was carried out using *Dynamics* software. 

### 3.3. Fluorescence Experiments

In order to determine the critical aggregation concentration we utilized the fluorescent properties of pyrene [36]. Pyrene was dissolved in 100% ethanol and then added to different concentrations of peptides. Pyrene concentration in all the samples was 2 μM. The concentrations of the peptides used were 0.5 μM, 1 μM, 5 μM, 10 μM, 50 μM and 100 μM. All dilutions were carried out in phosphate buffered saline (PBS). The excitation scans were recorded between 315 to 360 nm with the emission wavelength set at 393 nm. A PTI quantamaster instrument (Photon Technology International, Inc., Birmingham, NJ, USA), equipped with double monochromators, was used to record all the fluorescence measurements. We used 1 cm path-length cuvettes for all experiments and the sample volume was 1.2 mL. The slit width of the monochromators was set to 2 nm throughout all experiments.

### 3.4. NMR Experiments

The ^1^H-^13^C and ^1^H-^15^N NMR experiments were carried out on a 900 Mhz Bruker Avance Spectrometer (Bruker, Billerica, MA, USA). The spectrometer was equipped with a cryogenic probe. The buffer was 50 mM Tris-citrate pH 6.5, 50 mM NaCl, 5 mM MgCl_2_, 10 mM β-mercaptoethanol and 10 mM CaCl_2_ and the experiments were carried out at the temperature of 25 °C. Data analysis was done using NMRPipe. In case of the ^1^H-^15^N NMR experiments, the mean chemical shift difference was calculated using the following formula:

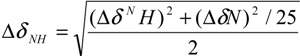
(1)


Statistically significant chemical shift differences were considered to be the ones higher than the sum of the average and one standard deviation.

### 3.5. Reductive Methylation of CaM

Reductive methylation of CaM was done using the technique described by Means and Feeney [37]. The procedure for carrying out reductive methylation of CaM has been previously described in our publication [23]. After carrying out reductive methylation on CaM, all the water in the buffer was replaced with D_2_O in order to minimize water signal.

### 3.6. Protein Purification

The plasmids were transformed into BL21AI cells. The cells were grown in LB media at 37°C for 15 hours and shaken at a 250 rpm with the use of kanamycin for antibiotic selection. Then the cells were transferred into M9 media with the dilution of 1:20 and grown at 37 °C and 250 rpm until the OD was 0.6–0.8 at 600 nm. Induction was carried out by adding 0.2% arabinose, 2% ethanol and 200 μM IPTG. After 4 h from induction, cells were harvested by centrifugation at 8000 rpm. The pellets obtained were lysed using 10 mM Tris-HCl, 1 mM CaCl_2_ and 10 mM β-mercaptoethanol at 60 °C for 1 h. The lysate was centrifuged again at 18000 rpm for 30 min. The pellets from this centrifugation were discarded and the supernatant was loaded onto a phenyl sepharose column pre-equilibrated with 10 mM Tris-HCl, 1 mM CaCl_2_ and 10 mM β-mercaptoethanol. The same buffer was used for washing the column loaded with supernatant until no more protein was detected by the Coomassie Plus Assay. The elution of the protein was done using 10 mM Tris-HCl, 5 mM EDTA, and 10 mM β-mercaptoethanol. Eluted fractions were assessed for purity using an SDS Page gel. Pure fractions were dialyzed against 50 mM Tris-citrate pH 6.5, 50 mM NaCl, 5 mM MgCl_2_, 10 mM β-mercaptoethanol and 10 mM CaCl_2_. A mass spectrum of purified CaM used for the experiments is provided in supplementary data section (Figure S1). The purified protein was concentrated using an Amicon pressurized stir-cell equipped with a filter membrane (EMD Millipore, Billerica, MA, USA) with a 10,000 Da cut-off whenever needed.

### 3.7. Modification of the Hypervariable Region

The HVR peptide with the sequence ‘KEKMSKDGKKKKKKSKC’ was purchased from the Protein Research Laboratory at the University of Illinois at Chicago. A solution of S-farnesyl-l-cysteine methyl ester in 100% dimethyl sulfoxide (DMSO) (73.6 mM, 68 µL) was added to no-weigh sulfosuccinimidyl-4-(*N*-maleimidomethyl)cyclohexane-1-carboxylate (sulfo-SMCC, 2 mg, Thermo Scientific, Waltham, MA, USA), and mixed thoroughly until all the sulfo-SMCC was dissolved. A solution of 50% (w/v) N-octyl-β-d-glucopyranoside (50 µL, Sigma Aldrich, St. Louis, MO, USA) was added to the above mixture, followed by PBS pH 7.4 (381 µL) and 50 mM di-*tert*-amyl peroxide (DTAP, 1 µL). The reaction mixture was incubated for 1.5 hours and thereafter diluted to the final volume of 8 mL with PBS. The solution was centrifuged at 4,500 rpm for 30 min at 4 °C. The supernatant was discarded and the pellet dissolved in 100% ethanol (150 µL) followed by the addition of 50% (w/v) N-octyl-β-d-glucopyranoside (100 µL). The above solution was added to the HVR peptide (2.0 mg) dissolved in PBS (750 µL) and incubated at room temperature overnight. The reaction mixture was then applied to a C-18 column and purified by reversed phase high performance liquid chromatography (RP-HPLC). Briefly, the column was equilibrated with 90% Buffer A (0.1% trifluoroacetic acid) and 10% Buffer B (90% acetonitrile) before applying the sample. Thereafter, a linear gradient up to 80% Buffer B was applied. The fractions eluted at 66–68% Buffer B were collected and subjected to mass spectrometric analysis for purity. The pure fractions were pooled and dried using an Eppendorf Microfuge. Since the peptide does not have aromatic residues the yield was estimated using a previously described protocol that takes advantage of the absorbance difference at 215 nm and 225 nm [38]. The schematic of the reaction for making FM- HVR is outlined in the supplementary data section (Figure S3). Mass spectra for both, the modified and the non-modified HVR, are also provided therein.

## 4. Conclusions

In conclusion, we used reductive methylation to enhance sensitivity of conventional NMR methods. Although FM-HVR aggregates beyond a concentration of 10 μM, we have successfully used NMR to study its interaction with CaM at very low concentrations by reductively methylating CaM. Thus, we have uncovered the interactions between CaM and the post-translationally modified HVR domain of K-Ras4B in the physiologically relevant low concentration range. This would have been difficult to accomplish without the use of the reductive methylation approach.

## Supplementary Materials

Supplementary materials can be accessed at:  http://www.mdpi.com/1420-3049/18/6/7103/s1.
