# Supplementary material for: Application of Reductive 13C-Methylation of Lysines to Enhance the Sensitivity of Conventional NMR Methods
Source: Molecules. 2013 Jun 18;18(6):7103–19. doi: 10.3390/molecules18067103 (PMC6270119; doi:10.3390/molecules18067103)

Supplementary Materials

Figure S1. Mass spectrum obtained after pooling the elution fractions of purified CaM.

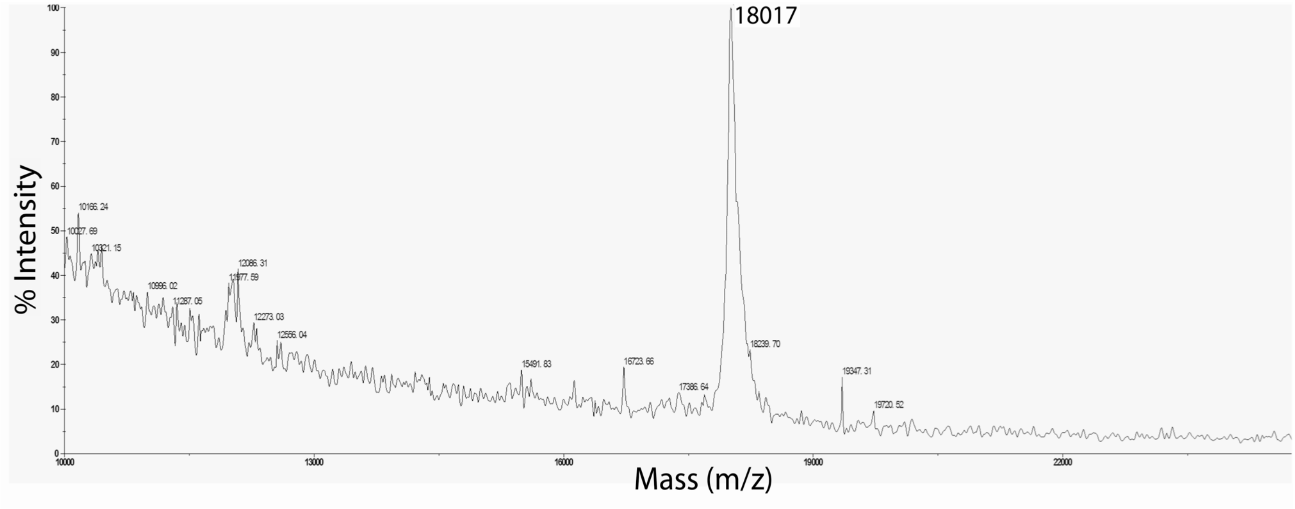

Figure S2. Mass spectra obtained before and after modifying the hypervariable region domain of K-Ras.

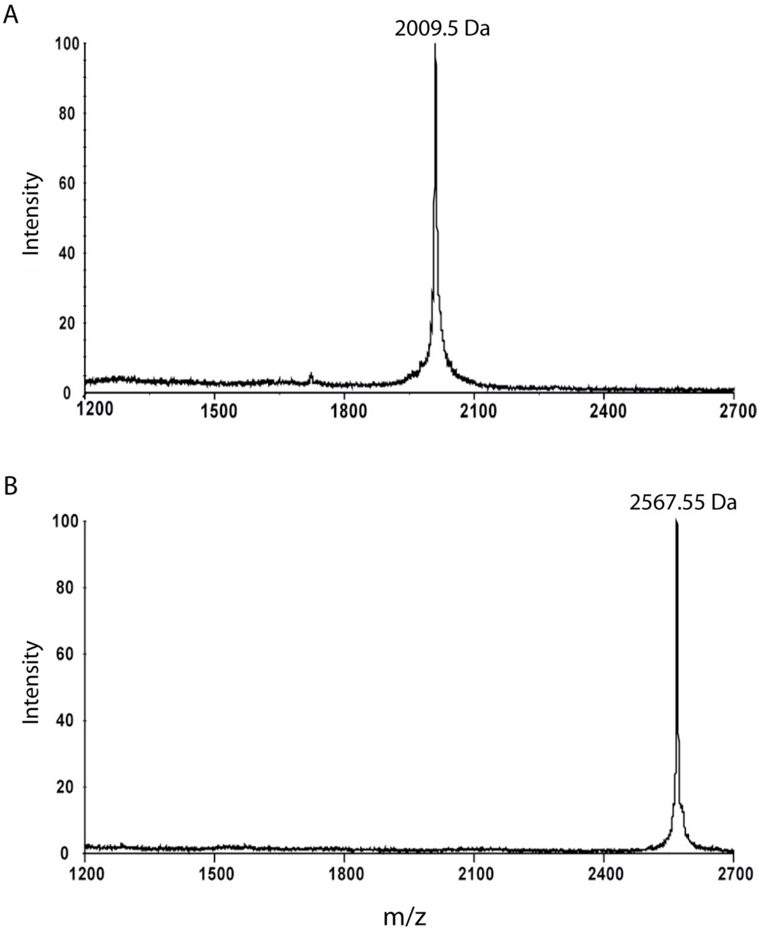

Figure S3. Reaction scheme for making farnesylated and methylated hypervariable region.

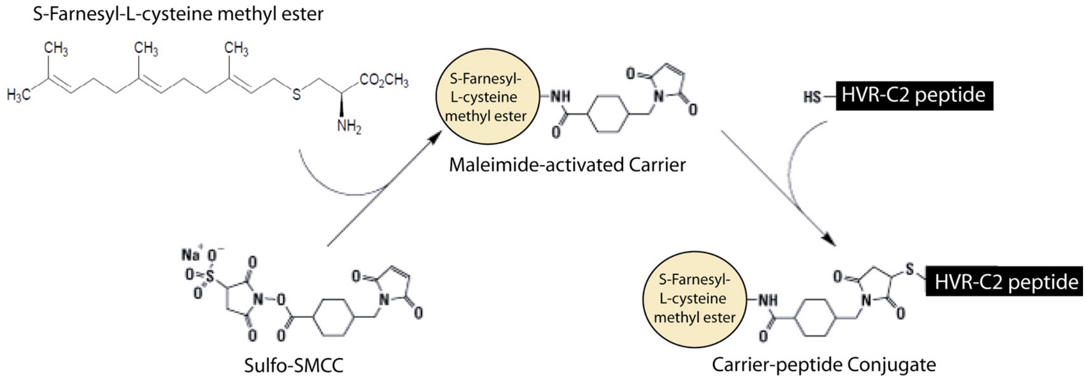

Figure S4. ITC isotherm obtained upon titrating 100 µM wild type CaM into 20 µM FM-HVR. The dissociation constant was 9.95 ± 0.15 µM. The data fitting was done using one set of binding sites model.

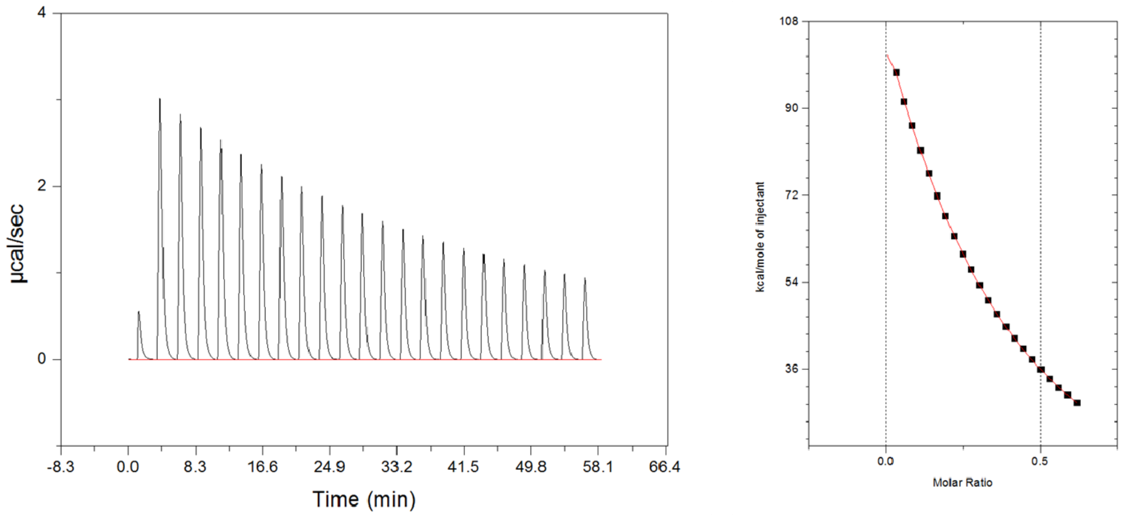

Figure S5. ITC isotherm obtained upon titrating 200 µM reductively methylated CaM into 20 µM FM-HVR. The dissociation constant was 9.42 ± 0.5 µM. The data fitting was done using one set of binding sites model.

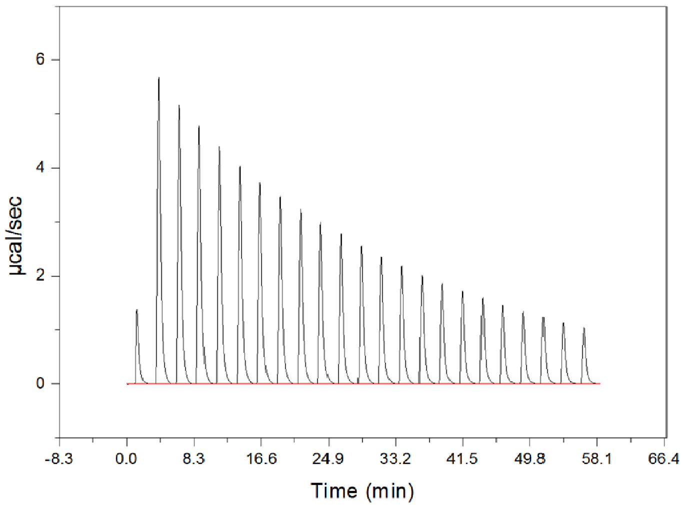

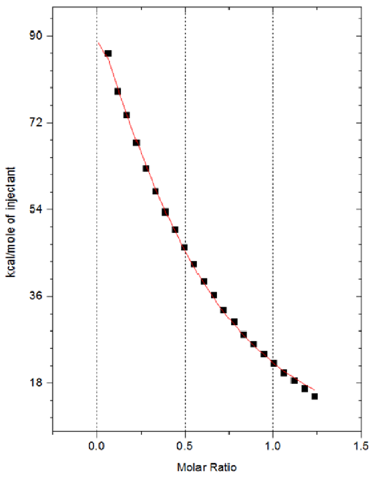

Figure S6. Positions of all lysines in CaM. Residues K94 and K75 are labeled accordingly (PDB: 1CFF). The NMR signals of these residues are shown closely in **Figure 5(b)** and **5(d)**, respectively.

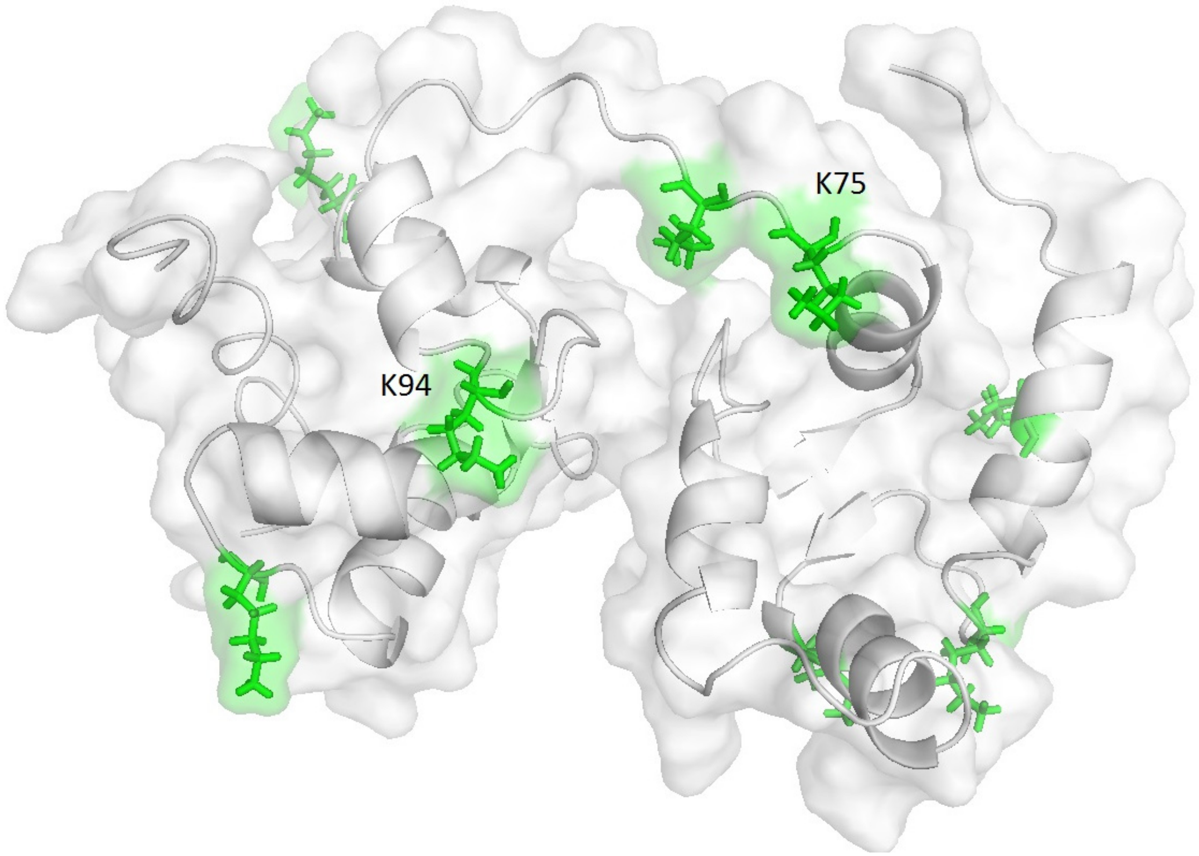

Figure S7. Example fluorescence spectra obtained from different concentrations of (**A**) HVR and (**B**) FM-HVR. The legend shows the respective concentrations of the peptides used. All samples contain 2 μM pyrene.

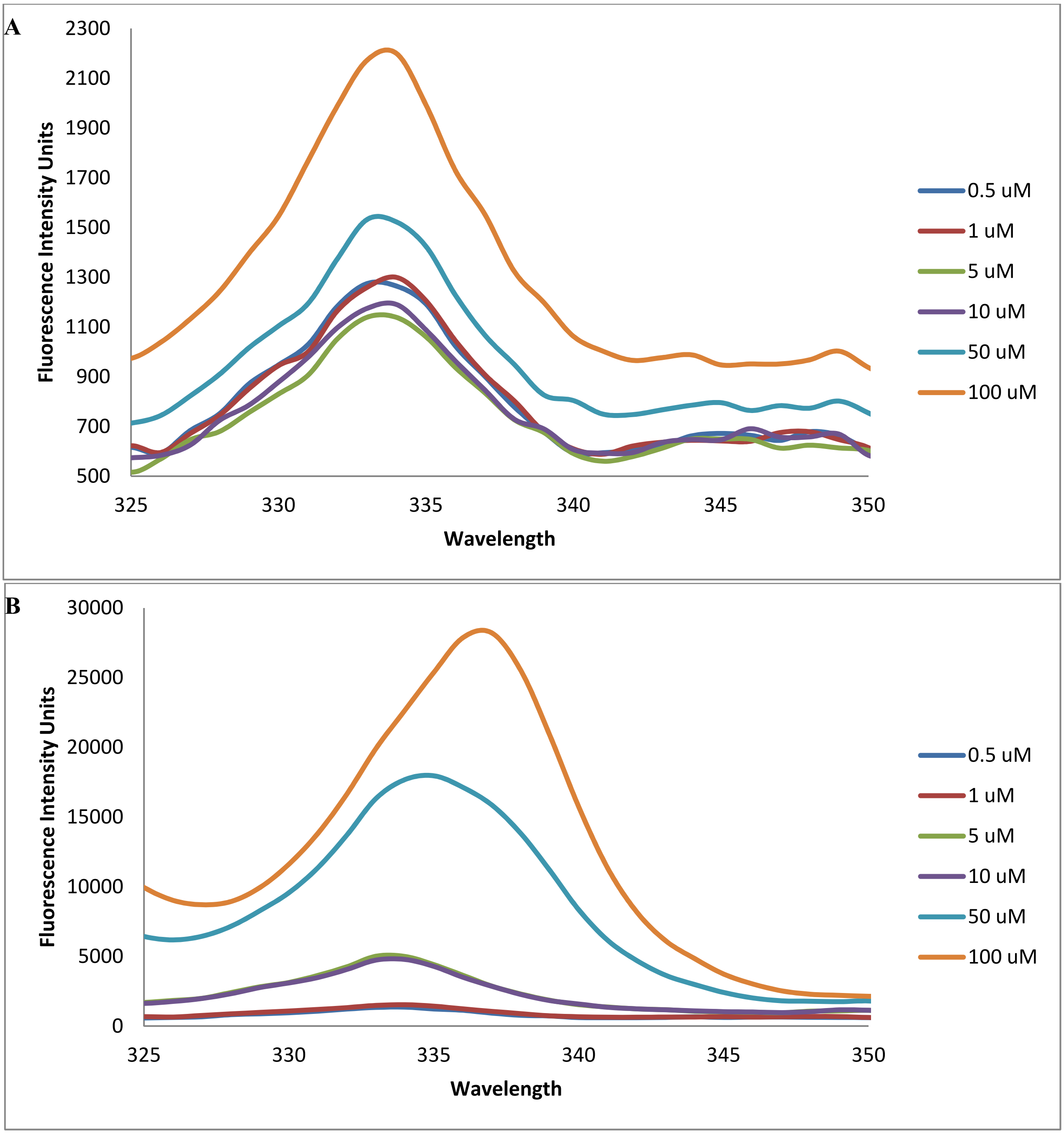

Supplement: Supplementary file 1 [file molecules-18-07103-s001.pdf]
